# Developing “My Asthma Diary”: a process exemplar of a patient-driven arts-based knowledge translation tool

**DOI:** 10.1186/s12887-018-1155-2

**Published:** 2018-06-05

**Authors:** Mandy M. Archibald, Lisa Hartling, Samina Ali, Vera Caine, Shannon D. Scott

**Affiliations:** 1 College of Nursing and Health Sciences, Flinders University, Sturt Road, Bedford Park, Adelaide, SA 5042 Australia; 2 Department of Pediatrics, Faculty of Medicine & Dentistry, University of Alberta, Edmonton Clinic Health Academy, 11405-87 Avenue, Alberta, T6G 1C9 Canada; 3 Faculty of Nursing, University of Alberta, Level 3, Edmonton Clinic Health Academy, 11405-87 Avenue, Alberta, T6G 1C9 Canada

**Keywords:** Asthma, Knowledge translation, Parents, Children, Family-centered, Arts-based knowledge translation, Storytelling, Intervention development

## Abstract

**Background:**

Although it is well established that family-centered education is critical to managing childhood asthma, the information needs of parents of children with asthma are not being met through current educational approaches. Patient-driven educational materials that leverage the power of the storytelling and the arts show promise in communicating health information and assisting in illness self-management. However, such arts-based knowledge translation approaches are in their infancy, and little is known about how to develop such tools for parents. This paper reports on the development of “My Asthma Diary” – an innovative knowledge translation tool based on rigorous research evidence and tailored to parents’ asthma-related information needs.

**Methods:**

We used a multi-stage process to develop four eBook prototypes of “My Asthma Diary.” We conducted formative research on parents’ information needs and identified high quality research evidence on childhood asthma, and used these data to inform the development of the asthma eBooks. We established interdisciplinary consulting teams with health researchers, practitioners, and artists to help iteratively create the knowledge translation tools.

**Results:**

We describe the iterative, transdisciplinary process of developing asthma eBooks which incorporates: (I) parents’ preferences and information needs on childhood asthma, (II) quality evidence on childhood asthma and its management, and (III) the engaging and informative powers of storytelling and visual art as methods to communicate complex health information to parents. We identified four dominant methodological and procedural challenges encountered during this process: (I) working within an inter-disciplinary team, (II) quantity and ordering of information, (III) creating a composite narrative, and (IV) balancing actual and ideal management scenarios.

**Conclusions:**

We describe a replicable and rigorous multi-staged approach to developing a patient-driven, creative knowledge translation tool, which can be adapted for use with different populations and contexts. We identified specific procedural and methodological challenges that others conducting comparable work should consider, particularly as creative, patient-driven knowledge translation strategies continue to emerge across health disciplines.

## Background

Educating parents about the complexities of childhood asthma is foundational to effective asthma management [1]. However, parents continue to struggle with fear and uncertainty about childhood asthma care, a problem compounded by inconsistent and often ineffective provision of asthma education [2, 3]. The paradoxical existence of high quality research evidence about asthma and continued deficiencies in parental knowledge, self-efficacy and suboptimal at-home asthma management is a knowledge translation (KT) problem. As such, finding effective ways to deliver asthma education to parents is essential given that asthma prevalence and childhood asthma morbidity continue to rise globally [4].

KT is an iterative process of synthesizing, disseminating and ethically applying knowledge to improve health, health services, and health systems globally [5]. Yet, mobilizing knowledge for use by health care professionals alone is insufficient to affecting change in real-life settings [6]. KT strategies targeting the wide-range of stakeholders (e.g., parents, non-health care professionals) are necessary, particularly in light of the growing emphasis on patient-centeredness, shared-decision-making, and community-based health management of chronic illnesses, including childhood asthma.

Arts-based approaches to KT offer viable ways of engaging key knowledge-users, such as parents, in a meaningful manner. Arts-based KT, defined here as the use of any art form to communicate knowledge (e.g., research from various sources), re-present and re-construct data, and promote empathetic understanding to affect attitudinal, knowledge or behavioural change, is gaining momentum across health disciplines concerned with mobilizing evidence to improve health outcomes [7, 8]. Arts-based approaches enable a human relationship to form with otherwise impersonal information by using artistic techniques, such as plot, characters, and specific vernacular [9, 10].

Although arts-based KT shows promise, the field is in an early developmental stage for healthcare professionals and data pertaining to the development, application, and evaluation of arts-based KT is generally lacking. As such, few examples of how to create arts-based KT strategies exist in the literature [11]. We address this gap by offering an account of how a visual art and story-based KT tool for parents of children with asthma was developed, while highlighting challenges encountered during this process. Although we developed this tool for parents of children with asthma, we believe our process can serve as a guide for others conducting similar work with different populations, including those interested in science communication (i.e., SciComm) more generally. Through describing this process we also explore general tensions related to the broader field of arts-based KT.

### Arts-based knowledge translation

Different artistic representations convey various expressive qualities and as such, foreseeably impact viewers in distinct ways [9]. Visual representations may foster more emotive than rational responses compared with text [12]. Music ignites the imagination and fosters the development of mental models to help make sense of the world [13]. Theatre promotes engagement and renders abstract concepts, concrete [14]. The selection of an arts-based approach should be informed by an understanding of the form; population, context, and location of use; desired outcome of interest (e.g., attitude change); and the degree of precision in key messages and extent of participation enabled through each form of representation [7].

Considering how to faithfully represent health research data and how to precisely convey key messages are paramount considerations in arts-based KT. Precision and communication accuracy may account for the absence of exclusively visual KT tools in the literature [15, 16]; text is a necessary accompaniment to the visual form if precision in information delivery, a critical aim of much KT, is to be attained [7]. These considerations should occur in tandem with the extent of participation enabled through the selected strategy; for instance, whether a knowledge user can help shape the outcome of a narrative, as is the case with forum theatre or classical “choose-your-own adventure” stories. Generally, health information interwoven into a printed story has less variability in its delivery; the precision in its key messages and the extent of participation with the art form are more consistent [7]. Although visual and text-based strategies tend to have limited interactivity, they are often portable and accessible, particularly when digitized. Artistic form and method of delivery thereby constrain and liberate aesthetic effects and foreseeably impact usability and effectiveness.

Information literacy theory supports combining textual and visual forms to depict information emotively and rationally [17]. However, the paucity of accompanying literature on developing these complex tools limits current understanding [12]. Specifically, visual and text can be combined in endless ways across artistic styles, methods of delivery, degrees of abstraction, precision of key messages and extents of participation (e.g., cartoons, poetry, interactive web-platforms) [7]. As such, identifying arts-based KT strategies with comparable attributes and purposes is particularly challenging.

Examples of visual and text-based tools for non-health care provider stakeholder groups can be found in the literature, yet reporting of tool characteristics are generally lacking (Archibald & Scott, unpublished observations). Lafreniere and colleagues [12] combined text and web-based cartoons to disseminate findings from nutrigenomics/nutrigenetics research. Each cartoon illustrated a research theme and was reinforced through text. The authors report that the approach was effective in conveying findings from the larger study, highlight pertinent procedural challenges to KT intervention development, and consider the impact of these challenges on effectiveness (e.g., simplification, aesthetics). Hartling and colleagues developed [11] paper-based storybooks for parents to communicate health information about childhood croup. Qualitative data from parents was used to revise the KT tools, led the authors to conclude that “the storybook format is a useful KT device” [18] (p. 162), and provided support for a user-centered development process. For our reported research, we learned from these examples, and designed a visual and text-based tool for parents, guided by parental information needs, preferences, and user-centered design principles.

## Methods

**Fig. 1:**
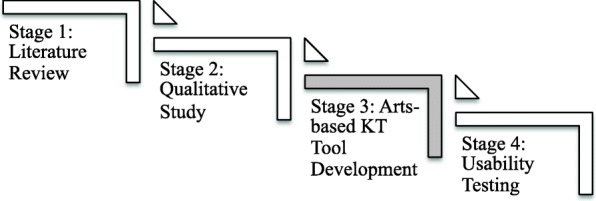
Four Stage Process for eBook Development

We used a four stage, iterative process to develop the asthma eBooks (Fig. 1). We have detailed stage one and the interpretive description component of stage two in previous manuscripts [3, 4] and therefore only briefly describe these stages here. We then focus on stage three—the process of developing the arts-based KT tool. Stage four involves usability testing of the arts-based KT tool and will be discussed in a forthcoming manuscript.

### Stage 1: Literature review

We conducted a state-of-the-science literature review of the information needs of parents of children with asthma [3]. This review illustrated a need to explicitly assess the information needs of parents of children with asthma. Based on findings from the 11 included articles, we constructed a parental information needs taxonomy, which included (i) asthma basics (e.g., basic pathophysiology), (ii) treatment modalities, (iii) coping, and (iv) medical expectations. We then used this to inform the development of a semi-structured interview guide for use in a qualitative study.

### Stage 2: Qualitative study

#### Interpretive description of information needs

We conducted an interpretive descriptive study [3, 19] of the information needs of 21 parents of children with asthma from diverse backgrounds and stages of asthma illness in an urban pediatrics centre in Western Canada [3]. Our research questions focused on parents’ information needs and the general experiences of having a child with asthma. These data were foundational to developing the arts-based KT tool. For instance, parental uncertainty surrounding day-to-day asthma management enabled integration of “real-life” examples into the tool.

**Fig. 2:**
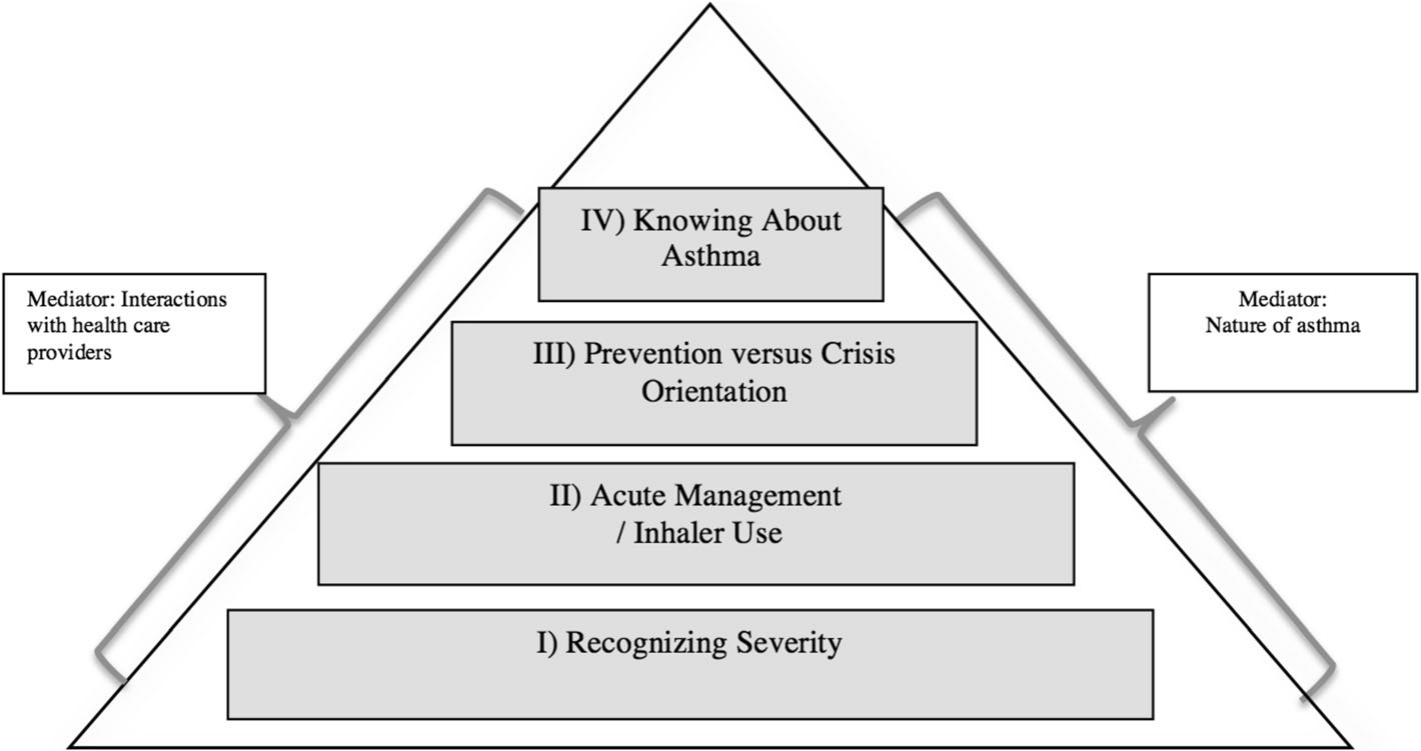
Hierarchy of Asthma Information Needs. From [2] *permission for reprint granted by Elsevier

Through thematic analysis we identified four core themes: (I) recognizing severity, (II) acute management and inhaler use, (III) prevention versus crisis orientation to asthma management, and (IV) knowing about asthma [3]. We identified interactions with health care providers (HCPs) (e.g., how education was provided) and beliefs about asthma (e.g., acute or chronic) as two factors influencing these themes. These themes formed an information needs hierarchy and influenced which information to include in the KT tool (Fig. 2).

### Stage 3: Arts-based KT tool development

**Fig. 3:**
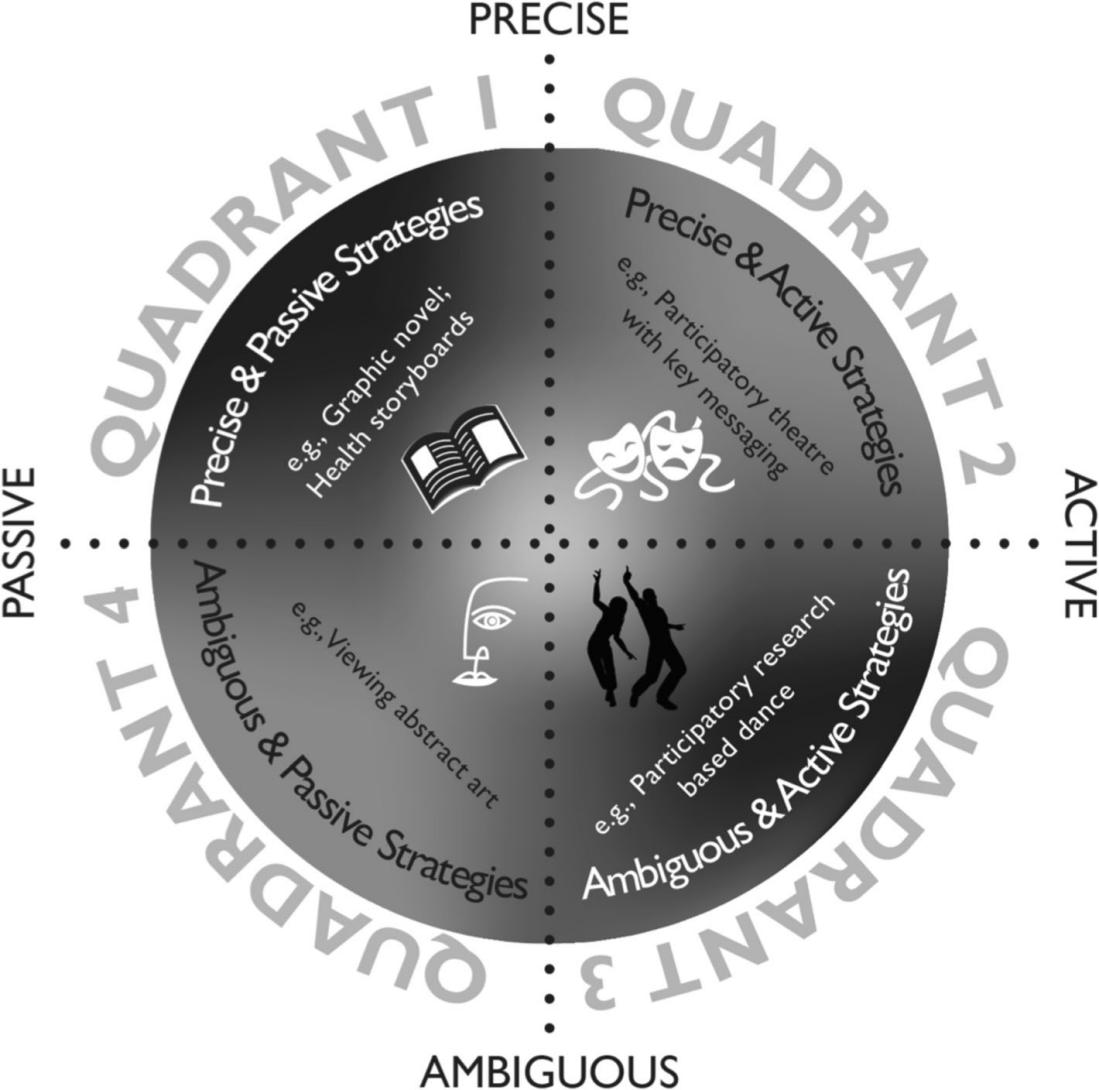
Classification Schema of Arts-Based Knowledge Translation Strategies. From [7] * permission for reprint granted by WILEY

We combined storytelling and visual art in an online format to deliver asthma education to parents. This decision was influenced by the stage two findings and the degree of ambiguity in key-message delivery permissible in this context of illness management [7]. For example, managing childhood asthma is a complex process involving viewing asthma as a chronic illness while responding appropriately during acute exacerbation periods. Integrating asthma management into family life, identifying and monitoring symptoms, and employing preventative measures are integral to positive outcomes. Factual and procedural knowledge related to asthma care are needed, which requires that information be clearly provided. Yet, changes in attitudes and beliefs about the nature of asthma are also necessary and may be less responsive to non-arts based information provision given the capacity of the arts to challenge entrenched assumptions [7]. The need to attend to knowledge, beliefs and attitudes reinforces the potential merits of using a story and visual arts-based KT approach. Using the Archibald Classification Schema of Arts-Based Knowledge Translation Strategies [7] (Fig. 3), we therefore categorized this strategy as a multimodal quadrant one approach due to the presence of precise key messaging and relative passive involvement with its use.

Determining the voice and presentation format for the tool were key considerations. A first-person, diary-format was selected for many reasons. First, participants emphasized the importance of personalized and compassionate education during the stage two interviews. A first-person narrative promotes resonance and authenticity in the reader, and is more personalized than a third-person approach. Second, in previous research, parents expressed a preference for the first-person over the third-person narrative [18, 20]. Third, the diary-format is aligned with the rise of the “reality phenomenon” prevalent in social cultures which leverages the concept of connectedness between viewers and characters [21]. A diary-format provides an insider’s perspective into the life of another family living with asthma.

#### Constructing interdisciplinary teams

We constructed two interdisciplinary teams to assist with developing the arts-based KT tool: (I) a review committee, and (II) a creative consulting team. The review committee consisted of five individuals with expertise in arts-based KT, visual arts, KT science, narrative methods, pediatrics, and emergency medicine. The team provided written or verbal feedback at various time points on versions of the illustrations and narrative. The in-person meetings indicated support for the KT approach and although conflicting perspectives arose, they resulted in important considerations about individual aesthetic preferences. In addition to the core review committee, a registered nurse at a participating pediatric asthma clinic provided clinical feedback when needed.

The creative consulting team was assembled to enable a mosaic of innovative ideas, capitalize on expertise in different styles of visual and narrative arts, and allow enough distance from the research team so that objective feedback could be provided. The first author advertised for a creative writer and illustrator on two freelancer forums, screened applications, and invited five visual artists to provide artistic samples based on an asthma-case scenario. One visual artist with experience in character development and diverse illustration styles was contracted. Similarly, the first author reviewed the curriculum vitae and writing samples of the creative writers; one writer was hired based on her extensive experience, enthusiasm for the topic, and perceived fit with the project and team. Confidentiality, work agreements, and terms-of-payment were agreed upon and signed. A digital media company was hired to digitize the KT tool for web hosting.

#### Developing the KT tools

The first author developed an outline of significant and common events in the experience of having a child with asthma, based on the frequency or impact of their occurrence during the stage two interviews. For instance, because receiving an asthma diagnosis was a noteworthy and anxiety-provoking event for most participants, these events were noted for the creative writer to include. “Illustration ideas” were included in the outline when appropriate but prescriptively directing the illustrator was deliberately avoided, as this would be antithetical to the creative generation possible through such a partnership.

The outline also included an “evidence insertion opportunities” column which listed information identified as important to parents and to the successful management of childhood asthma (Table 1). For instance, participants had concerns surrounding diagnosis. Similarly, parents had information deficits regarding the signs of asthma exacerbation [3]. Such incidences were classified as important educational opportunities based on their relevance to asthma management and the child’s well-being.

**Table 1:** Example of Asthma Diary Outline

Page #	Key Items for Narrative	Evidence Insertion Opportunities	Illustration Ideas
2	• Child showing asthma symptoms • Mother reflects on child’s frequent illness, including Emergency Department (ED) visits • Visits ED for respiratory illness • Multiple diagnostic procedures; experiences uncertainty • Receives asthma diagnosis	Asthma symptoms Incidence of viral infections Process of asthma diagnosis	Child in respiratory distress Child visiting ED with mother

The creative writer then crafted story entries to align with the outline and the first author ensured that evidence was included. The process of evidence insertion involved iteratively synthesizing and compiling diverse research (e.g., systematic reviews, qualitative studies, reputable websites, asthma guidelines) into a readable format to correspond with the emerging story framework. The “key items for narrative” column was refined and the creative writer drafted story segments that were reviewed to ensure the events, tone, and spirit of the participants’ experiences was reflected. Consulting with the review committee assisted when specific clinical questions arose.

In addition to synthesizing information, five links to existing educational online and community resources were provided. The host organizations were contacted in August 2014, and processes for requesting permission of content were completed. Linking to resources was important because locating and assessing information reputability is challenging [3, 22]. Additionally, parents felt that a list of resources would be useful during the previous information needs study and qualitative evaluation of croup storybooks [3, 11].

Although the research team and creative writer did not pre-determine the number of story entries, we did attempt to limit these to fewer than 25 for time and usability considerations. We felt that limiting the number of story entries prematurely could restrict the creative process. By entry number 18 we could foresee a natural end to the story and began tying together extraneous details. Once complete, the review committee ensured all textual information was clinically accurate, relevant, and readable.

**Fig. 4:**
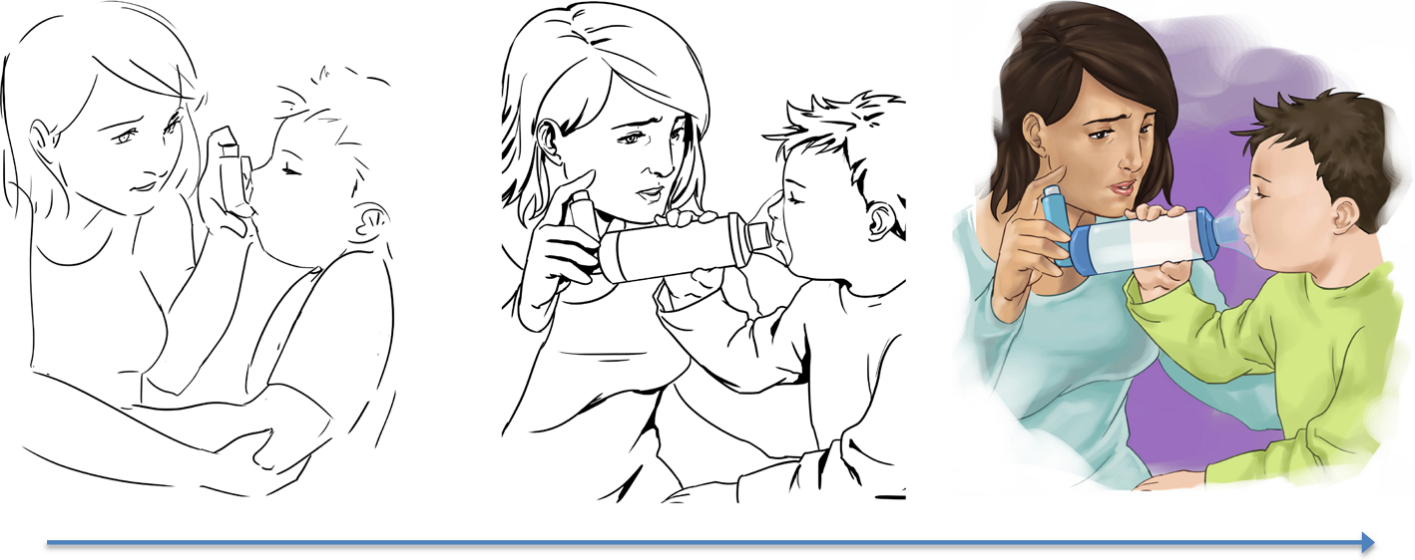
Iterations of Illustrations

The story segments were then provided to the illustrator who constructed visuals through a multi-stage process. First, she drafted an illustration to align with each story entry. These drafts were reviewed and suggested revisions were discussed with the review committee as needed. The illustrator revised all illustrations once the composition, feeling, and scene were agreed upon. The first author compared all illustrations to ensure internal consistency and requested further revisions. An average of three to four revisions per illustration was required before the final output was achieved. An example of the illustration iterations is provided in Fig. 4.

**Fig. 5:**
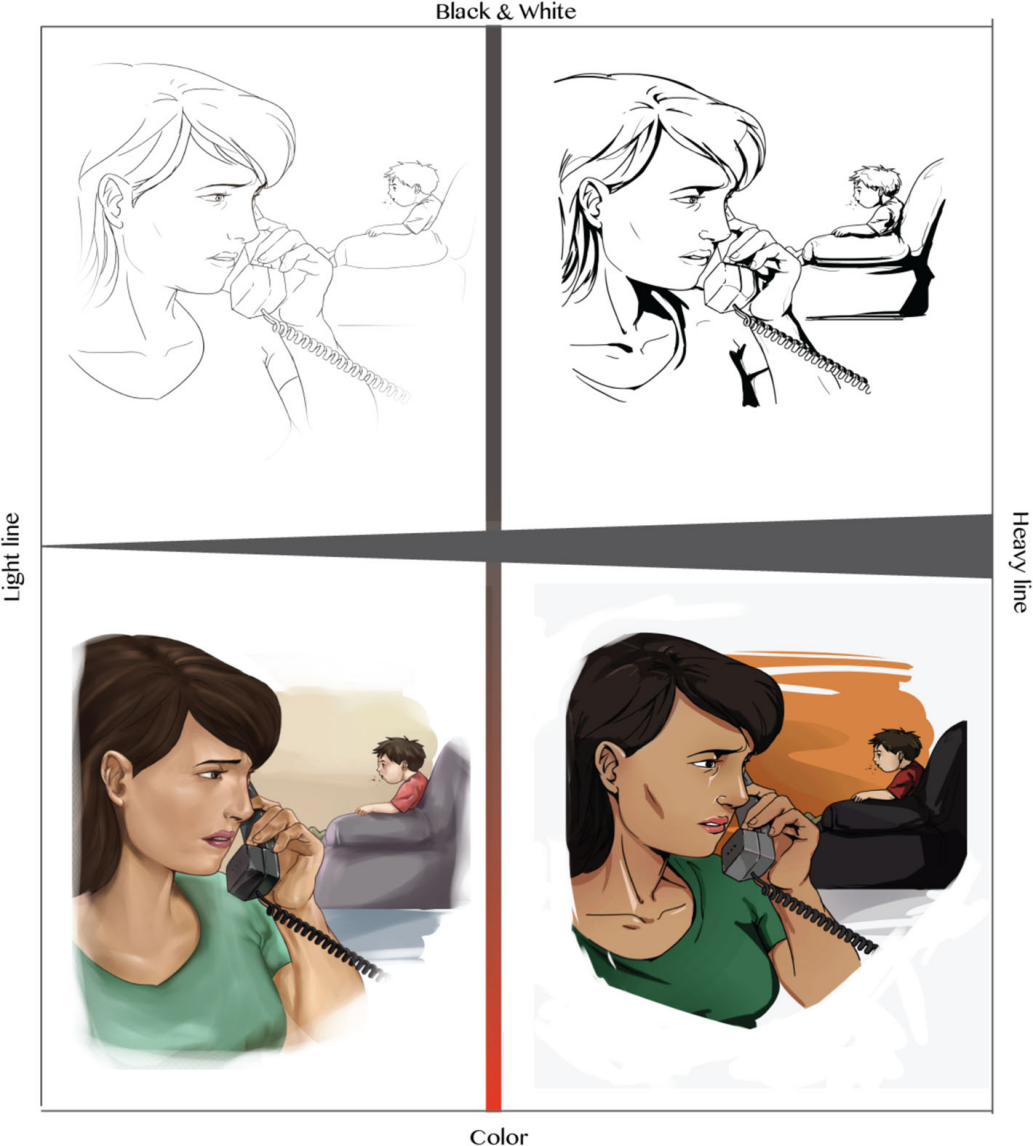
Arts-Based Knowledge Translation Prototypes by Line and Color Axes

We were interested in differences in parents’ responses to diverse illustration styles. As such, four distinct illustration styles were requested from the illustrator. The illustration styles differed by color and line; all other illustration components were unchanged across the prototypes (e.g., composition) (Fig. 5).

## Results

Although we previously reported on parents’ information needs from Stage Two, here we provide additional, previously unpublished data from the qualitative study on parent’s information preferences and then explore in the discussion how this helped inform the development of the KT tool.

### Topical survey of information preferences

Information about the format and perceived relevance of asthma education was essential to developing the KT tool. Although we depicted findings from the interpretive descriptive study largely at the level of thematic description (e.g., reflecting latent patterns), here we augment those results and provide previously unpublished findings to discuss the informational preferences of parents in a more concrete manner through topical survey [19]. During the interviews, we inquired about the information parents received, its effectiveness, and how they would like to receive information in the future. Parents generally felt they received insufficient information about asthma. Parents had difficulty identifying their information needs and numerous information deficits were present. For instance, when asked open-ended questions about asthma knowledge (e.g., What areas, if any, do you need more information about asthma?) parents infrequently identified areas of deficient knowledge about asthma or its management. The inability to identify specific learning needs limited parents’ information seeking behaviors at home and during interactions with HCPs. Further, many parents were overwhelmed by the abundant asthma information available online, and had difficulty determining its reputability [3].

When discussing their preferences about formats of information delivery, parents identified web-based information (44%) followed by pamphlets (33%) and face-to-face or verbal information (33%) as most desirable. Parents valued personalized information and reassurance provided by HCPs. Approximately 25% of participants commented that visuals, illustrations, or animation would be helpful ways of receiving information. Illustrating how an inhaler “works” on respiratory muscles or animating inhaler technique were examples of potentially useful visuals.

Emotionally sensitive information delivery was important to parents. Parents used the words “supportive,” “compassionate,” and “validation” to reference these emotions and at times desired more emotional support than they currently were receiving. As one parent stated “one thing I’d like. .. I don’t think I got much support. .. for the emotional side of it” (participant 18). Other participants emphasized the emotionally laden nature of having a child with asthma. As one mother stated, “it’s the emotional part that is the worst of it —it really is” (participant 7). Based on this, as well as the pervasive uncertainty, fear and anxiety expressed by parents during the interviews, we were attentive to the emotional sensitivity of the KT messaging. The emphasis on the emotional aspects of having a child with asthma, the desire for reputable web-based written information with visual components and the value placed on personalized information reinforced that an arts-based approach to KT may be a useful and effective method of delivering asthma education to parents.

We faced numerous challenges when developing the arts-based KT tool that may likely be encountered in future efforts. We summarize four of these considerations, including: (I) working within an inter-professional team; (II) quantity and ordering of information; (III) creating a composite narrative, and (IV) balancing the actual with the ideal, and later discuss approaches to mitigating these challenges. These challenges as experienced by the first author and KT development leader were documented as reflective memos throughout the process of tool development. Challenges were shared and discussed with members of the inter-professional team during face-to-face meetings, with particular debriefing and consultation occurring between the first and fifth authors.

#### (I) Working within an inter-professional team

An unanticipated challenge we encountered was the perceived appropriateness of feedback. Researchers may be accustomed to a high volume of feedback because of the culture surrounding grantsmanship, co-authorship, and peer-review. Artists may be less accustomed to this type of exchange. During our process, a collaborating artist expressed that her creative process was hampered by the detailed feedback received.

#### (II) Quantity and ordering of information

Information sequencing was an ongoing challenge. At times a concept (e.g., asthma action plan) was introduced in one diary entry but was not explained until later. Explaining each concept as it was introduced was not always feasible, particularly for recurring concepts (e.g., triggers) because other concepts (e.g., emergency asthma kits) were only present in one story segment and therefore took precedence. The challenge of information ordering raised potential issues for parents navigating the KT tool; the table of contents created did not align seamlessly with the information provided. Information was matched to the diary-entries wherever possible.

#### (III) Creating a composite narrative

Amalgamating the experiences of multiple participants into a composite narrative while maintaining resonance, appeal, and authenticity for the reader was one of the most profound challenges encountered. Commonalities between the experiences of multiple participants were easier to integrate into the overall storyline. Participant cases that were considered “outliers” were not directly included; rather, language reflecting the individual nature of children’s asthma trajectories was at times selected.

Terminology considerations were also ongoing when developing the composite narrative. We strived for relatability and aimed to use parents’ words whenever possible; however, parents used various terms to refer to health related concepts. For instance, parents referred to the emergency department as the emergency, emergency room/ department/ clinic/ facility/ ward and “ER,” with “emergency” being the most common term. The reviewing paediatrician (S.A) advocated for the more formal term “emergency department”; as such, we to had to balance parents’ terminology with that of practitioners who may be endorsing the tool.

Parents also used different terms to describe HCPs. The vast majority (86%) referred generically to “doctor” multiple times during an interview. Nurses were referred to less frequently (38%). No parent mentioned nurse practitioners or health care professionals. Emergency professional and HCPs were referenced by 5% of participants. We were alerted to the merits of specificity and consistency in terminology when the same parent would use multiple terms to describe the same concept.

#### (IV) Balancing the actual with the ideal

We grappled with authentically portraying parents’ experiences of care when they did not reflect ideal medical practices. For instance, some parents reported that HCPs reinforced the notion of growing out of asthma [3]. This reinforced beliefs about asthma as an acute condition and undermined the importance of prevention and day-to-day management. To address this, characters in the KT tool discussed their hopes about growing out of asthma but chronicity was reinforced.

As another example, mothers represented 95% of parents presenting for asthma related care in this sample. We reflected this in the storyline but not without hesitancy. We recognized that our sample might not represent the wider population of parents of children with asthma. Further, one parent found the overrepresentation of mothers in the croup storybooks to be an inaccurate depiction of real-life management scenarios [11]. We decided to portray a mother as the primary character in our story and included the father in the text and illustrations.

Balancing the ideal and the actual also related to the emotions of parents. Parents in Stage Two commonly experienced panic, anxiety, fear, and uncertainty. We needed to convey these experiences to enhance relatability but did not want to reinforce that parents should panic during asthma exacerbations. We were cognizant that doing so may contradict messages conveyed in the KT tool. This tension was captured in the comments of one expert reviewer: “*ran suggests urgency and panic*. *.*. *I would like to relay some comfort and decrease the panic*. *.*. *if the point is to mirror what a ‘real parent’ might feel at home, then the word is exactly right*!” Recognizing that different terminology serves different purposes, we felt compelled to stay true to the data in our narrative; parents in the qualitative study expressed urgency and we mirrored this through our selected terminology.

## Discussion

Our unique inter-professional team assembled in this study consisted of a nurse academic and artist, a creative writer, an illustrator, a digital media company, and an interdisciplinary review committee representing nursing, pediatrics, emergency medicine, and KT science. Through this study, we discovered the potential of collaboration to foster innovation and enable creative approaches to research problems, yet found that its success is greatly influenced by communication dynamics, mutual understanding, and the characteristics of individual collaborators [23, 24]. Challenges such as communicating across disciplines may be proportionate to the diversity of collaborating professionals [25]. Communicative openness and a willingness to receive feedback were imperative to overcoming these challenges. To overcome challenges faced by the quantity of feedback received by team members, the research team provided the artists only with feedback of a substantive nature and the first author (M.A) made editorial adjustments independently. Challenges with the quantity of feedback received were not encountered between the researchers and the digital media company.

Balancing information comprehensiveness with length was an ongoing consideration. Hartling and colleagues [11] encountered similar struggles; parents generally desired abundant information about croup (an acute respiratory illness) but some found the storybooks to be too lengthy. This challenge extends beyond the technical into the realm of aesthetics, highlighting a fundamental tension of using arts-based approaches for KT. Specifically, any extraneous inclusion detracts from the concision of the artistic rendering, which potentially reduces its aesthetic appeal, and foreseeably hinders its effectiveness [9, 10]. We mitigated this by limiting each diary-entry to approximately 90 words (a common length for a short paragraph), providing information in point-form when possible, externally linking to supplemental content, and eliminating content not immediately reflective of asthma priorities, parents’ information needs, and information deficits as identified in our previous research.

Participants’ ability to relate to characters was a recurring theme [11, 18]. We contend that this challenge of verisimilitude [10] is not unique to arts-based representations but reflects issues of representation more generally; there is a longstanding tension between representing individual cases alongside a shared reality. The tension between general knowledge and shared experience and between the individual experience and experience *applied* to the individual has also been illuminated by others exploring research methods [21, 26]. We recognized that we could not resolve this tension, yet we strived to create a narrative that reflected some aspect of the majority of parent’s experiences. To assist with this, we examined our data for negative cases/outliers. We determined that narrative examples from these outliers were less likely to resonate with the majority of parents and as such, were not directly communicated in the composite. To honor individual narratives, we selected examples from the lives of our participants and used their own words to convey these experiences. This reflects Denzin’s (2012) perspective that “our texts must always return to and reflect the words persons speak as they attempt to give meaning and shape the lives they lead” [26] (p. 5).

We grappled with using specific, consistent terminology that is valued in research or the diverse and often, nonspecific terms used by parents. Unfamiliar terms may lack appeal and alienate parents from diverse backgrounds. Specificity may be undesirable when parents possess low baseline knowledge about asthma. For example, parents generically referred to “pulmonary tests.” The reviewing paediatrician (S.A) questioned how specific terminology should be when providing information about this content area: “*there are pulmonary function tests*. *.*. *there are peak flow tests*. *.*. *do you want to label it more specifically, or are you being purposefully vague?”* In these instances we referred back to the participant interviews to address this question and found that 29% of participants referred to “pulmonary tests” in any capacity. Participants most often referred to “a test,” or, a “breathing” or “capacity” test. Only 10% of participants referred to a pulmonary function test and never to peak flow. Given this, the decision was made to use general terminology.

The issue of voice extended beyond using parents’ terminology in the diary entries. We questioned whether to present evidence as personal (e.g., “your child may. ..” ) or impersonal (e.g., “common symptoms include. ..” ). This was a challenge of evocation; that is, ensuring the work can reach the reader to arouse feeling, and therefore meaning [9]. A personal presentation of evidence was generally adopted for this reason.

## Conclusions

There is a need to learn about the processes and potential challenges associated with developing patient-driven arts-based KT tools, particularly as these strategies continue to emerge. Data is lacking on how arts-based KT resources have been developed, the process of integrating research evidence with artistic form, and associated challenges encountered. Working with stakeholder groups (e.g., through qualitative research) is necessary to identify the need for, and appropriateness of, an arts-based strategy. Foundational research can help identify which knowledge sources the target audience uses; preferences held about information delivery, and pervasive information and emotional needs. Fluency with the artistic form(s) and information literacy within the creative team are required to construct a meaningful and aesthetic artistic output with merit as a KT strategy. Establishing a review committee of individuals with clinical, content and methodological expertise is recommended to ensure accuracy and relevancy of the KT approach. We believe that collaboration within a diverse inter-professional team, considerations related to the quantity and ordering of information, representation issues encountered through creating a composite narrative, and harmonizing actual and ideal management scenarios are likely to be encountered when developing patient-driven arts-based KT tools.

## Abbreviations

ED: Emergency department
HCP: Health care provider
KT: Knowledge translation
